# Correction of Malocclusion by Botulinum Neurotoxin Injection into Masticatory Muscles

**DOI:** 10.3390/toxins10010027

**Published:** 2018-01-02

**Authors:** Hyun Seok, Seong-Gon Kim

**Affiliations:** 1 Department of Oral and Maxillofacial Surgery, Chungbuk National University Hospital, Cheongju 28644, Korea; sok8585@hanmail.net; 2 Department of Oral and Maxillofacial Surgery, College of Dentistry, Gangneung-Wonju National University, Gangneung 25457, Korea

**Keywords:** botulinum neurotoxin, masticatory system, maxillofacial bone, dental occlusion, orthognathic surgery, BTX injection into masticatory muscles affects the maxillofacial bony growth and dental occlusion. In clinical practices, BTX injection has been used for reducing post-operative relapse after mandibular surgery.

## Abstract

Botulinum toxin (BTX) is a neurotoxin, and its injection in masticatory muscles induces muscle weakness and paralysis. This paralytic effect of BTX induces growth retardation of the maxillofacial bones, changes in dental eruption and occlusion state, and facial asymmetry. Using masticatory muscle paralysis and its effect via BTX, BTX can be used for the correction of malocclusion after orthognathic surgery and mandible fracture. The paralysis of specific masticatory muscles by BTX injection reduces the tensional force to the mandible and prevents relapse and changes in dental occlusion. BTX injection in the anterior belly of digastric and mylohyoid muscle prevents the open-bite and deep bite of dental occlusion and contributes to mandible stability after orthognathic surgery. The effect of BTX injection in masticatory muscles for maxillofacial bone growth and dental occlusion is reviewed in this article. The clinical application of BTX is also discussed for the correction of dental malocclusion and suppression of post-operative relapse after mandibular surgery.

## 1. Introduction

For the correction of malocclusion, the understanding of growth and development is a key component. Broken harmony between the maxilla and the mandible during growth influences dental occlusion [1]. Most discrepancies in growth are under genetic control [2]. However, the interaction between muscles and the skeleton is the front line in the battlefield of growth. For these reasons, many orthodontists have used orthopedic appliances to correct abnormal jaw bone growth.

Dental occlusion is located in the border between the buccal shelf and the tongue. A patient with a narrow dental arch is treated using the appliance, which shields the action of the buccinator muscles. To prevent tongue thrust habit, an appliance can be used for treating anterior open-bite. In the case of pediatric mandibular prognathism, a chin cap, high-pull headgear, and other types of functional appliances have been used to restrain forward growth of the mandible. However, these efforts have often not achieved their treatment goals because most patients have shown relapse [3]. Compared to young patients, the results of orthodontic treatment are poor in aged patients because of slow bony remodeling and periodontal problems [4]. In the case of maxillary molar area, 20–30% of relapse has been reported between one and three years after treatment [5,6]. As the reasons of malocclusion are many, such as genetics, environmental and habitual factors, clinicians should consider all contributing factors [7]. Generally, relapse after treatment is associated with the severity of disease. Accordingly, intensive maintenance is required for patients needing large corrections [8].

If certain treatments can strengthen or weaken the power of specific muscles, they may replace unpleasant long-term usage of orthopedic appliances. Botulinum toxin is produced by bacterium and can weaken the power of specific muscles. If the rationale of the orthopedic appliance treatment is applied, BTX can be a highly effective treatment option for the correction of malocclusion-associated problems. Accordingly, recent knowledge regarding BTX on facial bone growth is reviewed in this article. Additionally, several frontier clinical applications of BTX are discussed.

Botulinum toxin-A (BTX) is a family of BTX and is most commonly used in clinical practice [9]. BTX reversibly reduces muscle activity and induces muscle paralysis by inhibiting the release of acetylcholine in presynaptic membrane of nerve terminal [10]. This treatment degrades synaptosomal-associated protein of 25 kDa (SNAP-25), which is required for acetylcholine secretion and release [11]. This paralytic effect of BTX has been used in various fields of oral and maxillofacial regions for the treatment of facial muscle spasms, muscle myalgia, temporomandibular disorder, masticatory muscle hypertrophy, and cosmetic purposes [12,13]. A therapeutic dose of BTX can be safely used in masticatory muscles with few complications [14].

The BTX injection in masticatory muscles influences balanced masticatory activity, including food mastication, swallowing, and breathing [15]. The BTX injection can disturb the balanced masticatory muscle activity and lead to masticatory muscle weakness and decreased mastication activity [16]. The paralytic effect of BTX in masticatory muscles influences maxillofacial bone growth when administered in the growth phase [17]. In animal studies, BTX injection in masticatory muscles has an effect on various portions of maxillofacial bone growth that are muscle-related areas and significantly decreased size and morphology [18]. The unilateral masticatory muscle weakness by BTX injection induces the maxillofacial bone hypoplasia and facial asymmetry [19]. The decrease of masticatory muscle function and bite force contributes to the changes of molar tooth eruption state and potentially affects dental occlusion [19,20].

In this review, we introduced the balanced masticatory muscle function in normal masticatory activity and the role of masticatory muscles in the masticatory system. We investigated the effect of the BTX injection in masticatory muscles on the changes of maxillofacial bone growth, dental eruption, and occlusion. We also suggested the therapeutic application of BTX for the recovery and correction of dental occlusion.

## 2. Balanced Muscle Power in the Masticatory System

The masticatory system is a complex functional unit composed of the maxillofacial bones, masticatory muscles, teeth, tongue, and temporomandibular joint (TMJ) [21]. This system is supplied by vascular and neuromuscular supports for function and activity [22], is controlled by the neurological system and cooperatively interacts with head and neck musculatures, ligaments, salivary gland, lips, palate, and cheek [23]. Mastication activity is highly organized and controlled neuromuscular activity that is integrated with the various masticatory system components [21]. The neurologically coordinated mastication system effectively regulates mandible movement and contracture of surrounding tissue [24]. The balanced muscle function and jaw movement contributes to normal masticatory activities, such as food intake, digestion, mastication, speech, and swallowing [15].

Mastication is a highly complex and organized neuromuscular activity involved with bone, muscles, teeth, and surrounding structure [22]. This activity requires the movement of jaw and TMJ, and masticatory muscle activity [21]. Balanced masticatory muscle activity is effectively regulated by the central nervous system [24]. The sensory input from the receptors in teeth, periodontal ligament, and TMJ is received to the brain stem and cortex through the afferent nerve [25]. The brain stem and cortex organizes this sensory input and provides motor activity output through efferent nerve fiber in masticatory muscles [24]. The integrated muscle functions are possible by the control of the central pattern generator (CPG) in the brain stem [24]. The masticatory CPG is located between rostral poles of trigeminal and facial motor nucleus and composed of several nuclei, such as nucleus ambigus, nucleus tracti solitary [26]. A neural signal from the hypothalamus activates neurons in the medulla oblongata through the nucleus tracti solitari and elicits masticatory activity [27]. The motor activity of CPG regulates the contracture of certain muscles and the relaxation of others [28]. The CPG regulates the rhythm and timing of muscle activity such that the activities of chewing, swallowing, and breathing can be effectively performed [23].

Mastication is rhythmic and repetitive chewing action and the beginning of digestion [24]. The muscles activated during mastication are temporalis, masseter, medial and lateral pterygoid, and suprahyoid musculatures [15]. These masticatory muscles are innervated with the trigeminal nerve and receive motor activity through the trigeminal motor nucleus [15]. Each of the masticatory muscles is attached on both sides of mandible and correspondingly activated according to the jaw movement and chewing phase [29]. In the opening phase of mandible, the inferior head of lateral pterygoid and digastric muscle are activated, and the digastric muscle acts in the rotation of mandible [30]. The temporalis, masseter, and medial pterygoid muscles involve the closing phase of mandible and act on clenching and chewing the food bolus [15]. Food mastication activity is also supported by the function of the lip, tongue, and buccinators [22]. The lip and perioral muscles involve the intake of food to the oral cavity, and maintain the sealing of mouth during mastication [31]. The tongue and buccinators contribute to effective chewing by repositioning food on the occlusal surface of teeth [31].

Normal mastication activity and masticatory muscle function can influence the maxillofacial bone morphology [32]. Mandible has symmetrical bone morphology, being a mirror image [27]. This bone connects with the cranial bone by the articulation of TMJ [29]. In addition, mandible can be moved by the symmetric movement of TMJ and activation of both sides of masticatory muscles [33]. Disruption of this harmonious masticatory muscle function and movement of mandible affects the symmetric growth of maxillofacial bones [34]. In functional matrix theory, maxillofacial mandibular bone growth can be affected by attached muscle activity and surrounding soft tissue [35]. The balancing of masticatory muscle function and activity can affect harmonious maxillofacial bone morphology, jaw movement, proper dental occlusion, and TMJ function [33,36].

## 3. Broken Balance Muscle Function by BTX Injection and Its Effects on Maxillofacial Growth

Balanced masticatory muscle function is closely related with maxillofacial bone growth and development [32,35]. Impaired masticatory muscle activity affects the reduced growth of the craniofacial bone structure [37,38]. Animal studies that have masticatory muscle hypofunction by soft food diet, muscle myotomy, and motor nerve denervation show reduced growth of maxillofacial bone [37,39,40,41]. Masticatory muscle hypofunction affects the bone mass, size, and length [42,43], and also affects the composition of the trabecular bone and thickness of the cortical bone [44]. The maxillofacial bone growth can be affected by the paralytic effect of BTX when it is administered in masticatory muscles [45]. BTX is a neurotoxin that reversibly reduces muscle activity without tissue damage [12]. BTX injection in masticatory muscle can disturb the balance and symmetric growth of maxillofacial bone in growing rats [36], and affects the change of craniofacial bone dimension and composition [18,46].

### 3.1. The Changes of Maxillofacial Bone Growth by BTX Injection in Masticatory Muscles

BTX injection in masseter muscles decreases muscle activity and affects the maxillofacial bone growth in animal studies [16,45]. Masseter muscle is attached to the zygomatic arch and inserted to the ramus and angle of mandible [47]. With the use of unilateral BTX injection in rabbit masseter muscle, the bone volumes of zygomatic and mandibular bone are significantly reduced [43]. In addition, with BTX injection in the masseter of growing rat, the mandibular length and ramus height are also significantly reduced (Figure 1 and Figure 2) [17]. The unilateral injection of BTX in masseter muscle induces the growth retardation of mandible (Figure 2) [17,18,48] and causes mandible deviation and facial asymmetry in adult rats (Figure 2c,d) [36]. The BTX injection in temporalis muscle also affects craniofacial bone growth. The temporalis muscle extends from the temporal bone and to the coronoid process of mandible [18]. Rats that received BTX in unilateral temporalis muscles had a significantly reduced skull base dimension [18], and the premaxilla, maxilla, and zygomatic arch dimensions were also decreased [18]. These previous animal studies show that the hypofunction of masticatory muscle by BTX injection affects the growth potential of the involved craniofacial bone and induces morphological changes in facial bone growth [17,43].

Masticatory muscle function has an important role in maintaining the bone density and quality of skeleton [49]. Decreased muscle function affects the bone metabolism and remodeling [50], and increases osteoclastic activity and accelerates bone resorption [51]. Muscle paralysis contributes to the disruption of bone homeostasis and leads to bone degradation and morphological changes [50]. Masticatory hypofunction with a soft food diet affects the internal bone structure of mandible in growing rats [44] and shows the thinner cortical bone and reduced bone density in the mandible ramus region [44]. These changes are also observed in the BTX application in masticatory muscle. The paralytic effect of BTX in masseter muscle influences the structural changes of mandible in rat [19]. The BTX-injected side of the mandible shows the significantly reduced bone mineral content and cortical bone thickness [19,52], and the proportion of the trabecular bone area is also reduced [19]. The rats that were BTX-injected in both masseter and temporalis show significantly reduced the trabecular bone in the alveolar and condylar bone region [53]. BTX injection in temporalis muscle of rat reduces the bone mineral density in the bones associated with temporalis muscle [46].

### 3.2. The Effect of BTX on the Growth of the Mandibular Condyle and Condylar Cartilage

BTX injection into the masticatory muscle may influence the growth of the mandibular condyle [54]. BTX injection into the masseter muscle in mice shows the significantly reduced condylar head width [55], and the distance between the medial and lateral margins of the condylar head is also significantly reduced in growing rats [43]. In BTX injection into the masseter and temporalis muscles, the bone volume is reduced in the BTX-injected side of the condyle [53]. The BTX injection into the masticatory muscle is also related to the bone density and quality of the condyle [53,54].

In animal research, trabecular bone density and condyle thickness are significantly reduced by the BTX injection into the masseter muscle [18], and the marrow cavity and trabecular spacing area are significantly increased [53,55]. The osteoclast activity and bone turnover are decreased in the subchondral area of the condyle [55], which suggests that BTX has sufficient paralytic effects to affect condyle development and induce bony hypoplasia or mandible deviation [17,18,43]. Masticatory muscle hypofunction induced by BTX injection not only negatively affects condylar growth, size, and volume, but also bone density and quality [55].

The detailed mechanism for the change of the condylar cartilage has been unclear. BTX injection of the masticatory muscle influences the temporomandibular joint via altering masticatory loading and causes structural and cellular changes in the condylar cartilage [48]. The growing rat, after receiving a BTX injection into the masseter muscle, shows significantly thinner condylar cartilage compared to the non-injected side [46]. This structural change of the condylar cartilage is associated with a decrease in cellular proliferation and division in the proliferative zone of the cartilage [55]. Unilateral BTX injection in the masseter muscle leads to an increase in the apoptotic process and a decrease in cellular proliferation in the proliferative zone of the cartilage [48]. Decreases in chondrocyte proliferation and proteoglycan secretion are observed in the BTX injection side of the cartilage [55]. Additionally, this change is a cellular response to the decrease in loading on the condylar surface [55]. This result indicates that the muscle paralysis caused by BTX injection may have an inhibitory effect on condylar cartilage proliferation [56].

### 3.3. The Effect of Masticatory Hypofunction by BTX on Dental Occlusion

Masticatory muscle hypofunction caused by BTX injection decreases the bite force and affects the dental occlusion and tooth eruption [19,20]. The masseter muscle volume and weight are significantly reduced by BTX injection [19], and the maximum bite force is also decreased [57]. The weakness of the bite force is directly related to the loading on the occlusal surface and the eruption state of the tooth [45]. In animal research, rats receiving BTX injection in the masseter muscle show decreased masseter muscle size and weight and overeruptions of the lower molars and incisors [19]. Furthermore, the maxilla and mandibular molar height are also increased after BTX injection into the masseter muscle [17]. Masticatory muscle weakness caused by BTX injection affects the tooth eruption state [19,20], and this tooth overeruption can affect the facial morphological changes, such as anterior open-bite, increased anterior facial height, and dolichofacial morphology [58,59].

## 4. Clinical Application of BTX

The application of BTX on the perioral area has been performed for cosmetic purposes or for the treatment of temporomandibular disorder [12]. Recently, BTX application has been tried to prevent post-operative relapse after orthognathic surgery. Post-operative relapse has been reported after orthognathic surgery [60]. The main reason for post-operative relapse is the memory of masticatory muscles in their preoperative position [61]. When muscles and connective tissues are extended by jaw bone movement, the stretch receptor will be activated and attempt to restore its original length [62]. Accordingly, the prevention of postoperative relapse has been designed to resist muscular tension.

Considering that post-operative relapse after orthognathic surgery is induced by the muscular tension, the strategy for reducing muscular tension can be an effective treatment option. In this aspect, BTX injection can be a solution for the postoperative relapse. Though the literature on this issue is scarce, there are several articles on the correction of open-bite after the treatment of trauma. The open-bite can be frequently found in bilateral mandibular angle fractures and the chin is depressed by the contracture of the digastric muscles [63]. Most patients can be corrected by open reduction and intermaxillary fixation. When patients do not receive the open reduction in time, reduced segments might be unstable due to the tensional force of the digastric muscles. Similar to BTX injection, radiofrequency therapy is also effective for reducing muscular power and volume. Targeting the anterior belly of the digastric muscle, the application of radiofrequency therapy is effective for correcting post-traumatic open-bite [64]. Based on this finding, similar cases have been treated by 20-unit BTX injections into the anterior belly of the digastric muscle [65]. When the patient is in the state of open-bite, the anterior belly of the digastric muscle receives the tensional force according to the counterclockwise rotation of the mandible in the course of treatment (Figure 3). Accordingly, the mandible has a tendency of clockwise rotation after reduction, and this mechanism will contribute to relapse after treatment. In fact, BTX injection into the anterior belly of the digastric muscle has been shown to be successful, and there has been no relapse after injection (Figure 4) [65]. As improper injection of BTX in the neck may induce such complications as dysphagia, the precise localization of injection site may be important to avoid these complications [66].

In the case of malocclusion patients, the anterior open bite has been frequently observed, and this protocol can be applied for these patients. The treatment of the open bite has been challenging because it has multiple etiological factors [67]. The open bite can be caused by the imbalance of the growth between the mandible and the maxilla, airway obstruction, para-functional habits, and trauma [68]. In the case of mandibular prognathism, approximately 30% of patients show an open bite [69]. These skeletal open-bite patients show clockwise rotation of the mandible and higher anterior facial height [70]. The patients with mandibular prognathism and open bite can be corrected by surgical treatment, and the mandible is moved backward and counterclockwise after the operation [71]. Postoperative anterior open bite is caused by unstable condylar position and muscular pull [72]. Postoperative anterior open bite after orthognathic surgery is a kind of relapse, and its rate has been reported at 10 to 15% [73,74]. Many kinds of modifications have been introduced for minimizing postoperative relapse. Overcorrection is overtreatment, rather than therapeutic movement, considering repositioning of jaw bones. Distal cutting of the mandibular proximal segment has been done to reduce the tension applied on the pterygomasseteric sling after the posterior movement of the mandible. If the surgeon modifies the design of osteotomy, the amount and the type of muscles attached to each sectioned bony segment can be changed. By adapting vertical ramus osteotomy design, postoperative relapse may be reduced [75]. Myotomy, as a preventive measure of the postoperative relapse, is an aggressive approach that targets the muscle directly. Most literature claims that these modifications have been successful in reducing postoperative relapse. However, cutting additional bone and myotomy have higher rates of complications, such as bleeding and nerve damage. A number of clinicians are concerned that the duration of the therapeutic effects of BTX is temporary. However, BTX application for the prevention of postoperative relapse can be promising, considering that the greatest amount of relapse (47.8%) has been observed during the early postoperative period [76].

“Deep bite” is the opposite of open-bite. The status of malocclusion has been frequently found in mandibular retrognathism [77]. For the surgical correction of this malocclusion, the position of the mandible usually moves downward and the myohyoid muscle receives tension [78] (Figure 4). Accordingly, relapse after treatment occurs at a high frequency, regardless of treatment protocol [79,80]. There has been comparative study on this issue. BTX has been given to the myohyoid muscle to reduce tension after surgery [60]. When compared to untreated control, the BTX application group has shown significantly higher positional stability. Myotomy for the suprahyoid muscles also showed an increase in stability after the mandibular advancement, and these findings can be interpreted in that the tensional force of the suprahyoid muscles is a contributing factor for skeletal relapse [61]. Considering the complications of suprahyoid muscle myotomy [81], BTX injection is a relatively simple and effective treatment.

In the course of orthognathic surgery, patients usually prefer the intra-oral approach to the trans-buccal approach. However, compared to bi-cortical screws fixation, single plate fixation is less rigid fixation [82]. Patients who received bi-cortical screw fixation may open their mouth immediately after operation. In the case of single plate fixation, patients may be asked about intermaxillary fixation for three to four weeks. In our preliminary study [83], the patients (*n* = 7) received BTX-A injection into their masseter muscles along with two weeks of intermaxillary fixation. This group was compared to the patients (*n* = 11) who did not receive BTX treatment and the same period of intermaxillary fixation. The incidence of plate fractures was 14.3% in the BTX-injected group and 31.8% in the untreated control group (Figure 5). As the plate fracture is largely a fatigue type of fracture induced by the action of the masticatory muscles, reduced muscle power by BTX application may prevent the plate fracture. Though postoperative relapse has not been assessed, it may be reduced by BTX injection. To draw definite conclusions, further follow-up studies will be required.

The application of BTX in pediatric patients has been rare. In the progress of growth, the size of muscle fibers increases [84]. In experimental research, BTX prevents the exercise-induced increase in muscle fiber size of young rats via reduction in contractile activity [85]. When the upward movement of the maxillary posterior teeth is affected by posterior impaction, overbite in the anterior teeth can be increased, and anterior open bite can be corrected [86]. Accordingly, posterior bite block can be considered nonsurgical treatment of open bite [87]. If the open bite is caused by tongue thrust habit, tongue spurs can be used to control the force generated by tongue muscles [88]. When the parafunctional habit is intervened in the early stage of growth, irreversible open bite can be prevented [89]. The correction of open bite in the children is mainly composed of functional appliance that can shield the action of perioral musculatures [89]. Though many types of functional appliances have been introduced, their therapeutic effects have not been promising due to study design limitations [90].

BTX injection into perioral muscle has been considered a relatively safe treatment. Except for periocular injection, complications related to BTX injection have been rarely reported. Recently, a case of temporary blindness has been reported after BTX injection into the masseter muscle [91]. The blindness after BTX injection may be induced by intravascular introduction of BTX [92]. When BTX is introduced into the vessel, it may induce myocardial infarction and pulmonary embolism via pro-thrombotic effect [93]. When an ocular event is observed after BTX injection, early injection of steroid may be helpful for relieving retinal pressure [94]. To avoid intravascular introduction of BTX, BTX should not be diluted too much and a small-sized needle should be used. Deep injection may increase the probability of intravascular introduction of BTX. There is no difference in the therapeutic effect between intradermal and intramuscular injection of BTX [95]. To prevent systemic effects of BTX injection, the clinician should make every effort to minimize diffusion and vascular introduction of BTX after injection.

## 5. Conclusions

Balanced masticatory muscle function is a key component of maxillofacial bone growth and development. The dysfunction of masticatory muscle influences the retardation of facial bone growth and disruption of dental occlusion. The BTX injection to the masticatory muscle induces reversible paralysis and weakness of muscle power. The injection of BTX in masticatory muscle disrupts the balanced function of mastication and can influence maxillofacial bone growth and dental occlusion when administered during the growth phase. The weakness of masticatory muscle activity by BTX induces the hypoplasia of maxillofacial bone in the zygoma, temporal bone, mandible, and condyle area, and the alteration of the tooth occlusion state.

Clinically, the paralysis of masticatory muscle by BTX has an effect on maintaining mandible stability and preventing changes in dental occlusion after orthognathic and mandible fracture surgery. BTX injection in digastric muscle reduces the tensional force of mandible and prevents the counterclockwise rotation of mandible and open-bite of teeth. The BTX injection in mylohyoid muscle also prevents the deep bite of teeth and postoperative relapse after orthognathic surgery. Compared with a surgery-only patient, the patient in our clinic who received BTX in both masseter muscles after orthognathic surgery showed more stable dental occlusion. The BTX injection is an effective method for the correction of dental occlusion by inducing specific masticatory muscle paralysis without major complications. In an animal growth study, the injection of BTX in masticatory muscle has an effect on the growth potential of the maxillofacial bones. Additionally, this treatment could be an effective tool for the correction of facial bone and dental occlusion in the pediatric patient. Further study will be necessary for the therapeutic use of BTX in orthopedic treatment to correct abnormal jaw bone growth and malocclusion.

## Figures and Tables

**Figure 1:**
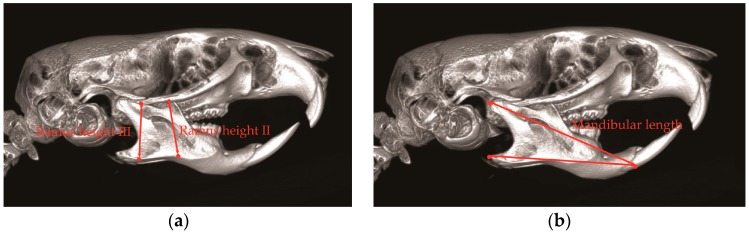
Anthropometric measurement of ramus height and mandible length. (**a**) Ramus height II is the distance between the zygomatic arch and inferior point of antegonial notch; ramus height III is the distance between the temporozygomatic suture of zygomatic arch and inferior point of mandible; (**b**) Mandible length is the distance between posterior point of mandible condyle and anterior point of mandible crest in mandible incisor.

**Figure 2:**
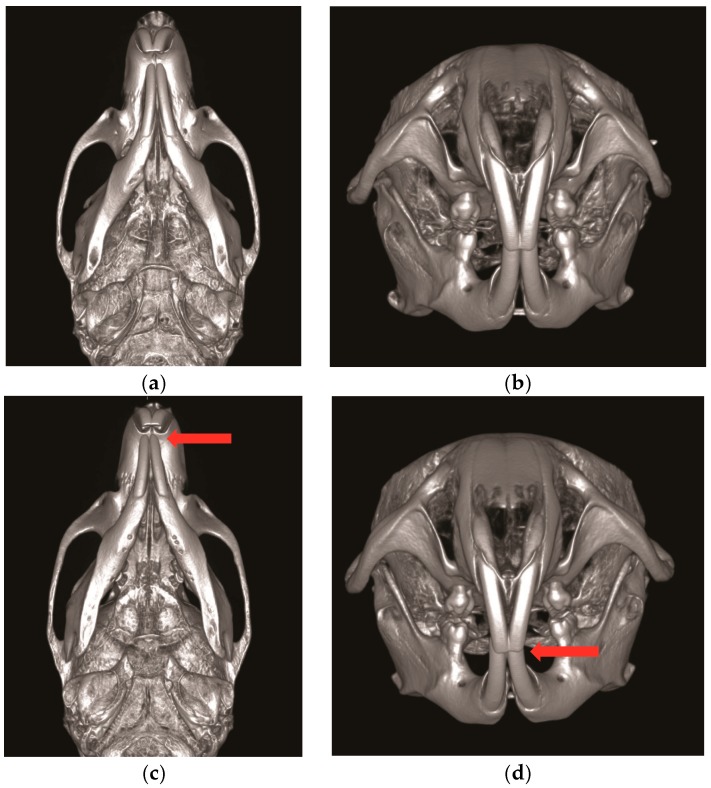
Unilateral Botulinum toxin (BTX) injection to the right masseter muscle induces the retardation of mandible and facial asymmetry. (**a**,**b**) The control group with saline injection to the right masseter muscle; (**c**,**d**) the experimental group with BTX injection to the right masseter muscle (red arrow; the deviation of the mandible midline to the BTX injection side).

**Figure 3:**
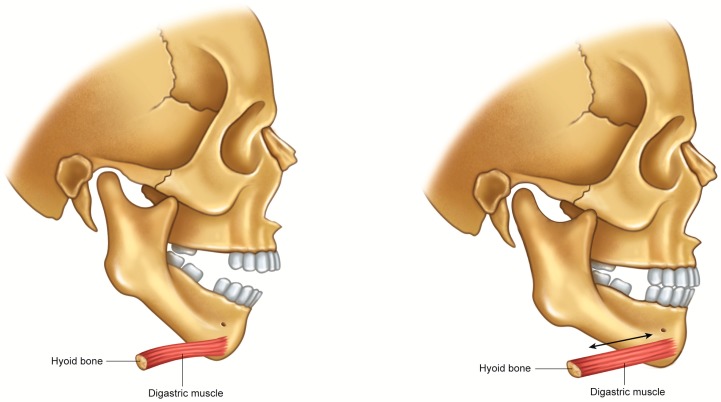
Schematic illustration of relapse mechanism after open-bite correction. During the correction of anterior open-bite, the mandible was rotated in a counterclockwise direction and the anterior belly of the digastric muscle was lengthened. Accordingly, the tensional force was generated and the relapse of the open-bite could have occurred during relieving the tensional force.

**Figure 4:**
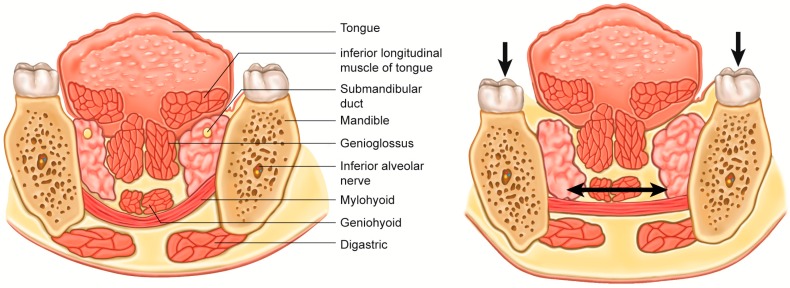
Schematic illustration of relapse mechanism after deep bite correction. During the correction of deep bite, the mandible was moved in a downward direction and the mylohyoid muscle was lengthened. Accordingly, the tensional force was generated and the relapse of the deep bite could have occurred while relieving the tensional force.

**Figure 5:**
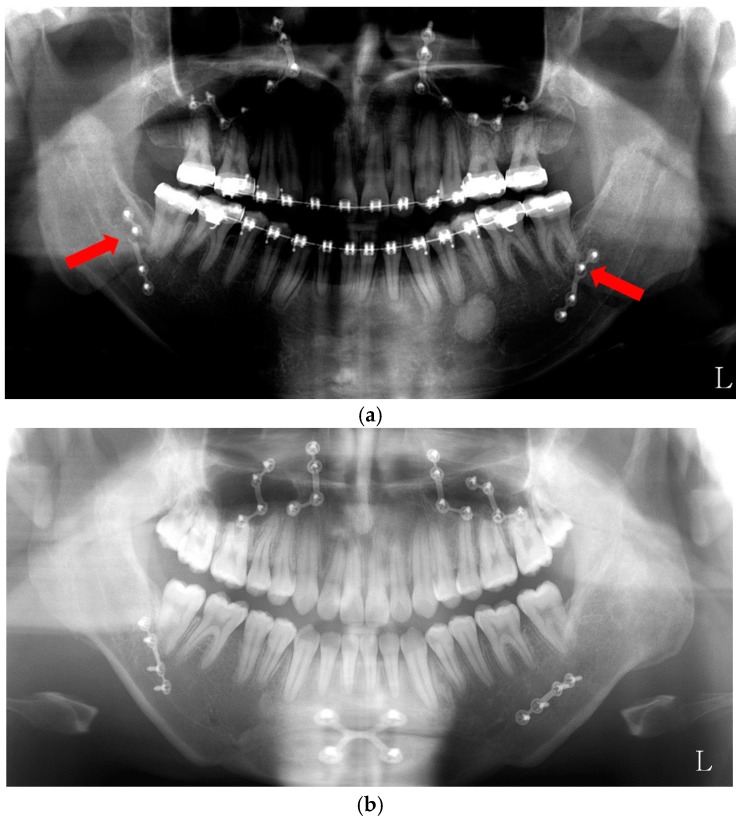
BTX treatment after orthognathic surgery. (**a**) Single plate fixation group after orthognathic surgery without BTX treatment (plate fracture in red arrow); (**b**) BTX injection group in both masseter muscles after orthognathic surgery.
